# The Length of an Infarcted Lesion Along the Perforating Artery Predicts Neurological Deterioration in Single Subcortical Infarction Without Any Relevant Artery Stenosis

**DOI:** 10.3389/fneur.2020.553326

**Published:** 2020-09-29

**Authors:** Seong Hwa Jang, Sang-Won Park, Doo Hyuk Kwon, Hyungjong Park, Sung-Il Sohn, Jeong-Ho Hong

**Affiliations:** ^1^Department of Radiology, Samsung Medical Center, Sungkyunkwan University School of Medicine, Seoul, South Korea; ^2^Department of Neurology, Keimyung University School of Medicine, Daegu, South Korea; ^3^Department of Neurology, Daegu Fatima Hospital, Daegu, South Korea

**Keywords:** infarct, neurological deterioration, stenosis, biomarker, stroke

## Abstract

**Objectives:** This study aimed to assess image biomarkers of early neurological deterioration in single subcortical infarction (SSI) without any relevant artery stenosis.

**Methods:** Between June 2005 and December 2009, consecutive patients with SSI within 24 h of symptom onset were enrolled. Magnetic resonance angiography of the brain and neck was obtained from all patients to confirm the absence of any stenosis of relevant arteries. We defined early neurological deterioration (END) as neurological worsening by ≥ 2 points based on the initial National Institutes of Health Stroke Scale score during the first week post admission or prior to hospital discharge. A multiple logistic regression analysis was used to evaluate the independent predictors of END in SSI.

**Results:** A total of 205 patients (109 males; aged 63.9 ± 11.0 years, range 39–90 years) were enrolled, of whom 158 (77%) remained stable or improved, while 47 (23%) showed neurological worsening. There were significant differences in the maximum diameter of the largest area on an axial view and in the number of slices showing cerebral infarction on a transverse plane between patients with and without END. A adjusting for age, hypercholesterolemia, hemoglobin, NIHSS on admission and these magnetic resonance imaging characteristics, the occurrence of having three or more slices showing the cerebral infarction on a transverse plane was an independent predictor of END in SSI without relevant artery stenosis (1 vs. 3; OR 14.281; 95% CI 1.76–115.8; *p* = 0.013, 1 vs. 4; OR 14.04; 95% CI 1.65–119.57; *p* = 0.016).

**Conclusion:** The longitudinal length of the infarcted lesion along the perforating artery predicts END in cases of acute SSI without any relevant artery stenosis.

## Introduction

Single subcortical infarction (SSI), or lacunar infarction, has a fair clinical outcome; however, previous studies have reported early neurological deteriorations (END) in 20–43% of SSI, leading to poor functional outcomes (1–3). Predicting whether an acute SSI will progressively worsen based on individual characteristics and brain imaging data during initial diagnosis helps to determine treatment strategies. However, SSI classifications have yet to be established and the mechanisms underlying END in patients with acute SSI remain to be elucidated. Hemodynamic factors, thrombosis extension, excitotoxicity, and inflammation have all been proposed as possible mechanisms of this progression (4–9).

SSIs are usually caused by small vessel disease or steno-occlusion of the orifice of a perforator at the parent artery (10). Branch-atheromatous disease (BAD) presents with different arterial pathologies compared with small vessel disease. Atheroma typically affects vessels proximally. In a recent study, END incidence was found to be significantly greater in BAD subjects (11). Local thrombosis superimposed on the ostial atheroma and thrombus propagation from either proximal to distal or distal to proximal segments of a perforating artery, along with progressive occlusion of lateral branches, may be the most plausible mechanism proposed (9). Although a previous study proposed that the term “small subcortical infarction” defines small (<20-mm transversal diameter) lesions in the territory of a penetrating arteriole, it does not imply any specific etiopathogenesis (12). Previous studies have also reported that middle cerebral artery (MCA) plaques are present in 42 to 60% of patients with lacunar infarction (13, 14); however, differentiating between BAD and small vessel disease in patients with no relevant artery stenosis on magnetic resonance-angiography (MRA) remains challenging and SSIs based on size remain controversial (15–17).

Therefore, we sought to investigate the biomarkers that affect END in acute SSI patients without any relevant artery stenosis, regardless of the infarct size.

## Materials and Methods

### Subjects

**Figure 1:**
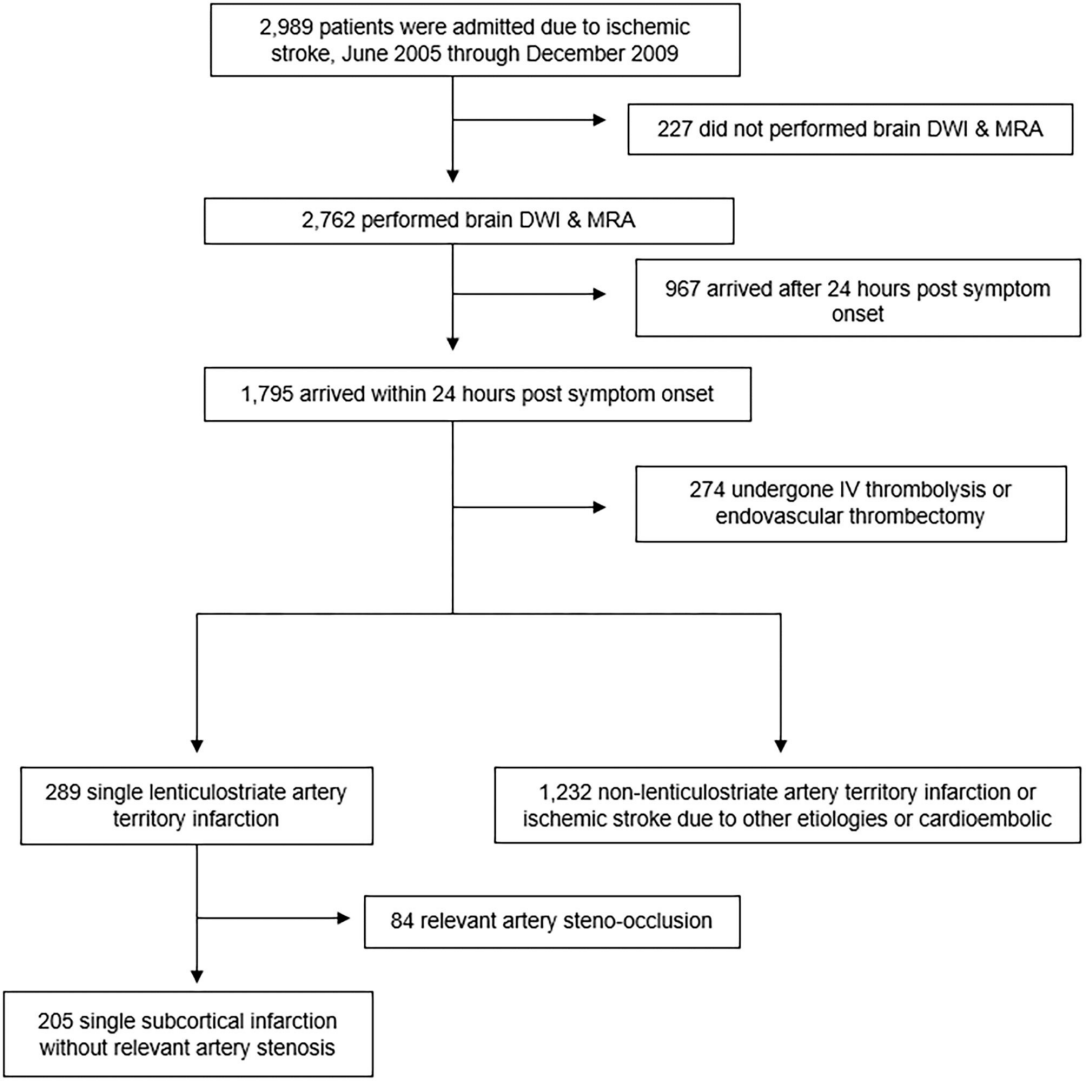
Study Flowchart. DWI, diffusion-weighted imaging; MRA, magnetic resonance angiography.

The retrospective analysis in this study was based on the Dongsan Stroke Registry, an ongoing single-center prospective cohort study of patients with acute ischemic stroke presenting at the Keimyung University Hospital in the Republic of Korea. All patients admitted with ischemic stroke from June 2005 to December 2009 were included in the study review (*n* = 2,989). We had collected analyzable cases using the following inclusion criteria: (1) ischemic lesions documented by brain diffusion-weight imaging (DWI) (*n* = 2,762) and (2) arrived within 24 h of symptom onset (*n* = 1,795). Patients who had undergone intravenous thrombolysis or endovascular treatment (*n* = 274) were excluded from the study; a total of 1,521 patients had met our study criteria. We confirmed that patients had a single lenticulostriate artery territory infarction without cardioembolic source such as atrial fibrillation and other etiologies such as cancer-related stroke, dissection, Moyamoya disease, or vasculitis (*n* = 289). After excluding 84 patients due to relevant artery steno-occlusion, a total of 205 patients were eligible for analysis (Figure 1). Acute stroke management was performed according to the current clinical practice guidelines (18) in which clinicians can decide whether to use dual antiplatelet therapy. Antihypertensive therapy is not used in cases of no hemorrhage risk during the acute stage.

### Imaging Analysis

All patients underwent DWI, brain magnetic resonance-imaging (MRI, Signa VH/I, General Electric, Milwaukee, WI), and MRA covering the cerebral arteries and neck within 24 h of symptom onset. Brain MRIs and MRAs were performed using a 1.5 or 3.0 Tesla scanner, while DWI parameters [repetition time of 7,500 ms, echo time of 84 ms, matrix number of 128 × 128, and 2 b values of 0 and 1,000 s/mm^2^] included a slice thickness of 5 mm and an inter-slice gap of 2 mm. Contrast-enhanced MRA was conducted to obtain carotid artery image in the neck. Two trained neurologists (S-I.S. and S-W.P, kappa score = 0.91) who were blinded to the clinical information assessed the degree of stenosis, the maximum diameter of the largest area on an axial view, and the number of axial image slices showing cerebral infarction. Any disagreement was resolved by re-evaluation after discussion. SSI was considered present when the high signal intensity on DWI was visible in the area of the lenticulostriate artery, regardless of the maximal lesion size on the axial DWI.

### Neurological Deterioration Definition and Data Collection

We evaluated the following clinical characteristics and laboratory parameters from the Dongsan Stroke registry: age, gender, hypertension (receiving antihypertensive medication or blood pressure > 140/90 mmHg at discharge), diabetes mellitus (prescribed diabetes medication, fasting blood glucose > 126 mg/dL, 2 h plasma glucose > 200 mg/dL, or HbA1c ≥ 6.5%), hypercholesterolemia (prescribed a cholesterol-reducing agent, fasting low-density lipoprotein > 160 mg/dL, or fasting total cholesterol > 240 mg/dL) (19–21), medical history of a previous stroke, cigarette smoking, onset-to-emergency room visit time, initial random blood pressure, leukocyte count, blood hemoglobin, platelet count, C-reactive protein (CRP) levels, D-dimer levels, and albumin levels. Echocardiography and/or Holter monitoring were conducted to detect the cardioembolic source in patients with large infarction (> 20 mm) according to the SSS-TOAST classification (22).

We assessed daily neurological status from admission to discharge using the National Institutes of Health Stroke Scale (NIHSS) score. END was defined as worsening by ≥ 2 points in the total NIHSS score or ≥ 1 point in the motor items of the NIHSS score compared to the initial NIHSS score during the first week of admission or before hospital discharge. We also reviewed the modified Rankin Scale (mRS) score at 90 days to evaluate clinical outcomes. This study was approved by the local Institutional Review Board of our hospital (IRB number 2020-05-021).

### Statistical Analysis

Patient characteristics, including vascular risk factors, laboratory parameters, NIHSS scores, and MRI findings, were compared between groups with and without END. In the univariate analysis, variables between the two groups were compared using a Student's test and a Mann–Whitney *U*-test for continuous variables. A Fisher's exact test was used to analyze categorical variables. A multiple logistic regression analysis was used to evaluate the independent predictors for END in acute SSI and was performed in a logistic regression model in which the entry was set at a univariate association of *p* < 0.2. These results were expressed as odds ratios (ORs) of the relative risk, with 95% confidence intervals (CIs). *p* < 0.05 was considered significant. All statistical analyses were conducted using SPSS (version 19.0; SPSS Inc, Chicago, IL, USA).

## Results

**Figure 2:**
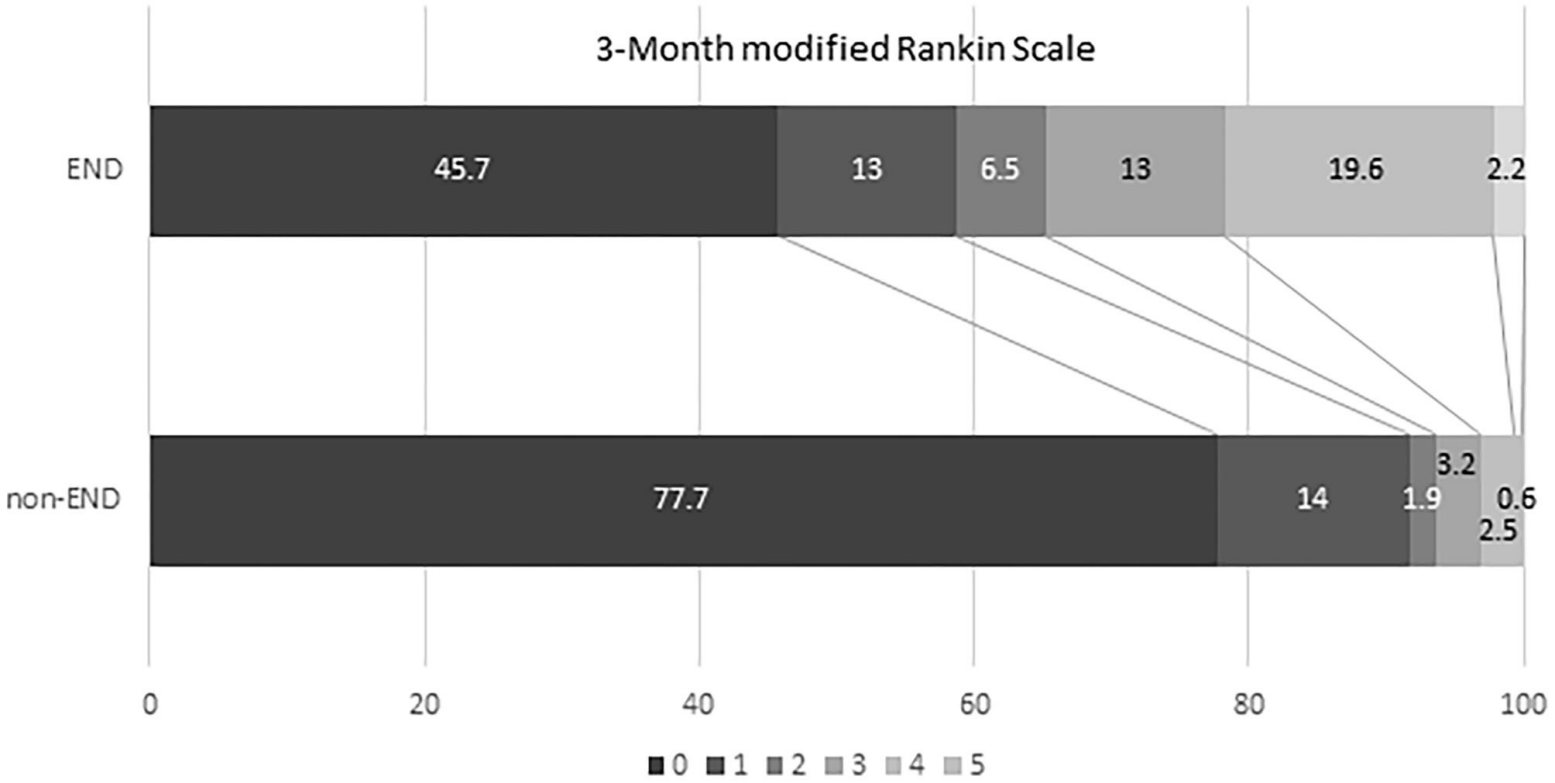
Comparison of clinical outcome (mRS 90 days) between non-early neurologic deterioration and early neurological deterioration groups. mRS, modified Rankin Scale; END, early neurological deterioration.

Over 4 years, 205 consecutive patients with acute SSI were identified. Patient characteristics of the selected study population are summarized in Table 1. Forty-seven (22.9%) of the 205 patients were diagnosed with END. Age, sex, presence of vascular risk factors, and laboratory parameters were not significantly different between both groups. There was also no significant difference between the two groups in the time it took patients to visit the emergency department following symptom onset. However, we found a tendency toward a significant difference in hypercholesterolemia (*p* = 0.076), NIHSS on admission (*p* = 0.083), and hemoglobin (*p* = 0.058) between the END and non-END groups. Functional disability reflected in the distributions of the mRS scores at 90 days was higher in patients with END than in patients without (Figure 2). The maximum diameter of the largest area on axial DWI and the number of axial image slices showing the cerebral infarction were both higher in the END group than in the non-END group, based on brain imaging data.

**Table 1:** Demographics and radiologic results in patients with and without neurologic deterioration.

	**No progression (*n* = 158)**	**Progression (*n* = 47)**	*p*-value^*^
**Clinical characteristics**			
Age, years	63.3 ± 10.7	66.0 ± 11.7	0.138
Male, *n* (%)	87 (55.1%)	22 (46.8%)	0.319
Hypertension, *n* (%)	112 (70.9%)	33 (70.2%)	0.929
Diabetes mellitus, *n* (%)	64 (40.5%)	20 (42.6%)	0.802
Hypercholesterolemia, *n* (%)	31 (19.6%)	4 (8.5%)	0.076
Previous stroke, *n* (%)	23 (14.6%)	6 (12.8%)	0.757
Smoking, *n* (%)	58 (36.7%)	15 (31.9%)	0.547
Onset-to-ER visit time (hr)	9.25 ± 6.7	9.32 ± 6.9	0.948
NIHSS on admission	3.22 ± 2.6	3.94 ± 2.2	0.083
**Laboratory parameter**			
Leukocytes, /μℓ	7,723 ± 1,979	7,286 ± 2,254	0.200
Hemoglobin	13.9 ± 1.6	13.4 ± 1.5	0.058
Platelet	263 ± 73	253 ± 76	0.391
CRP	0.31 ± 0.7	0.43 ± 1.0	0.397
Albumin	3.92 ± 0.59	3.81 ± 0.76	0.319
D-dimer	0.51 ± 0.62	0.49 ± 0.41	0.819
Random glucose	142.2 ± 59.3	149.3 ± 74.1	0.505
**MRI characteristics**			
Maximal diameter on axial DWI, *n* (%)			0.007
<15 mm	110 (69.6%)	21 (44.7%)	
15~20 mm	34 (21.5%)	17 (36.2%)	
>20 mm	14 (8.9%)	9 (19.1%)	
DWI slice No., *n* (%)			<0.0001
1	33 (20.9%)	1 (2.1%)	
2	52 (32.9%)	7 (14.9%)	
3	41 (25.9%)	21 (44.7%)	
≥ 4	32 (20.3%)	18 (38.3%)	
NIHSS at 7 days or discharge	1.91	5.15	<0.0001
mRS at 30 days	0.34	1.30	<0.0001
mRS at 90 days	0.41	1.54	<0.0001

*n, number of patients; ER, emergency room; NIHSS, national institutes of health stroke scale; CRP, c-reactive protein; MRI, magnetic resonance imaging; DWI, diffusion-weighted imaging; mRS, modified rankin scale*.

* *Based on χ^2^-test and student t-test*.

Multivariate analysis (Table 2) showed that the number of DWI axial image slices showing the infarct (3, OR, 14.28; 95% CI, 1.76–115.78; *p* = 0.013) and (≥ 4, OR, 14.04; 95% CI, 1.65–119.57; *p* = 0.016) was a significant independent predictor of END after adjusting for age, hypercholesterolemia, hemoglobin, NIHSS on admission, and the maximum diameter of the largest area on axial DWI. However, the maximum diameter of the largest area on axial DWI was not significantly associated with END.

**Table 2:** Independent predictor associated with neurological deterioration in multiple logistic regression analysis.

	**OR**	**95% CI**	***p*-value**
Age, years	1.02	0.99–1.06	0.22
Hypercholesterolemia, *n* (%)	0.41	0.13–1.32	0.13
Hemoglobin	0.91	0.72–1.15	0.44
NIHSS on admission	1.01	0.88–1.17	0.87
Maximal diameter on axial DWI (<15 mm)			
15~20 mm	1.41	0.60–3.30	0.43
>20 mm	1.59	0.53–4.78	0.41
DWI slice No. (1)
2	4.49	0.52–38.76	0.17
3	14.28	1.76–115.78	0.013
≥4	14.04	1.65–119.57	0.016

*NIHSS, national institutes of health stroke scale; DWI, diffusion-weighted imaging; No., number*.

## Discussion

In this study, we evaluated image markers predicting END in patients with acute SSI and no visual evidence of steno-occlusion of the relevant arteries. Although a previous study has reported that enlargement of the infarct volume may contribute to neurological deterioration in small vessel disease (2), it will require a long period and it is difficult to use in a clinical setting. Therefore, we assessed the number of image slices showing the infarct on axial DWI, which was an independent factor, to evaluate the vertical extension of the lesion and the thickness of the infarcted lesion. Furthermore, unlike other studies, we showed that the maximum diameter of the largest area on axial DWI was not a factor affecting END since we only included patients who had acute SSI with no visual evidence of any artery stenosis on the MRA. Relevant artery stenosis is a known risk factor for END (23). In addition, although Berbeich et al. suggested that older age and male sex were significantly associated with END (24), these two variables were not different between END and non-END groups in our study.

Several studies have examined the relationship between END and MRI findings in SSI (1–3, 9, 15, 25, 26). Importantly, Nakamura et al. (1) reported that lacunar stroke patients with END, based on T2WI findings, exhibited larger lesions than those without deterioration, while Terasawa et al. (2) reported that the initial infarct volume and enlargement of the infarct volume contribute to END. Moon et al. (26) found that an initial infarct extent of ≥ 15 mm was associated with motor symptom progression, and Jeong et al. (27) documented that patients with SSIs with relevant artery stenosis and branch atheromatous lesions are more likely develop END. Most previous studies conducted their analyses using the classic definition of SSI, in which stenosis is considered in relevant arteries if it has resulted in ≥ 50% stenosis of the lumen.

Traditionally, small subcortical infarctions were considered a category of small vessel disease, caused by the lipohyalinosis or fibrinoid necrosis of small arteries or arterioles extending to deeper structures (28). BAD was first described in 1989 as an alternative mechanism to lipohyalinosis. BAD vascular lesions are located more proximally along the perforator artery than lipohyalinosis. Thus, the ensuing ischemic lesion may be larger in BAD-related infarcts than in lacunar infarction (29). However, there is little consistency with regard to the dimensional cutoff accepted as the definition of a BAD-related infarct. In most studies, these lesions were considered identifiable by a diameter > 15 mm on axial DWI (30). In our study, the appearance of the infarct on three or more consecutive slices from axial DWI images was associated with END in patients whose relevant arteries had no evidence of stenosis on MRA. This suggests that longitudinal damage to small penetrating arteries contributes to END in patients without any relevant artery stenosis, unlike those with relevant artery stenosis, which may be explained by the fact that a longer lesion meant that patients had a long thrombus, making it possible to extend both retrogradely and anterogradely (31).

Stenosis in intracranial arteries such as the MCA is best detected via noninvasive methods such as MRA and CTA. Although distal subtraction angiography (DSA) is the golden standard, it is too invasive to screen for stenosis following ischemic events in patients with no visual evidence of stenosis in any relevant arteries. In a previous study, MRA was found to be equivalent to DSA in detecting > 50% of stenosis cases, with sensitivity, specificity, and accuracy of 92, 91, and 91%, respectively (32). We used MRA to detect relevant artery stenosis. However, diagnosis of arteriopathy linked to BAD using imaging techniques such as MRA or CTA is limited since these imaging techniques only depict the external morphology of arteries and are unable to show inner vessel wall changes. Recently, 3 Tesla high resolution (HR) MRI has been used to visualize the inner MCA. Chung et al. (13) performed 3 Tesla HR MRI scans on 15 patients with acute small subcortical infarctions and no relevant vessel disease identified on MRA (MCA or BA) and identified a relevant branch atheromatous plaque in nine subjects. In other words, patients without any relevant artery stenosis on MRA may also have BAD characteristics. A recent study showed that the incidence of END was significantly greater in a BAD group (11). However, it is not possible to perform HR MRI in all subcortical small infarction patients without any relevant artery stenosis. We used classic brain imaging tools such as MRI, MRA, and DWI and found that the longitudinal length of the infarcted lesion, as determined based on the appearance of an infarct lesion in ≥ 3 consecutive slices on a transverse plane, was significantly associated with the occurrence of END. Using modern techniques to measure brain perfusion (computed tomography or MRI), few studies have documented that perfusion deficits are associated with END (33–35). This result is consistent with the fact that perforating arteries have poor collateral connections, called end arteries, and are vulnerable to drop in blood pressure. Treatments to improve local perfusion, such as induced hypertensive therapy, are an important patient management strategy. One retrospective study found that phenylephrine-induced hypertension can result in early motor restoration without serious side effects in progressing SSI (36). A multicenter randomized clinical trial showed that phenylephrine-induced hypertension was safe and resulted in early neurologic improvement and long-term functional independence in patients with non-cardioembolic stroke. In subgroup analyses, treatment-induced hypertension had stronger effects on small vessel occlusion compared to larger artery occlusion resulting from stroke (37).

Our study has some important limitations. First, we included patients admitted within 24 h of stroke onset; therefore, progression prior to admission was not assessed. Second, we only enrolled Korean patients; thus, ethnic disparities were not considered. Third, since we used classic MRA to detect relevant artery stenosis, patients with relevant artery stenosis may have been misclassified as patients without relevant artery stenosis. We could have detected relevant artery stenosis had we used HR MRI. Fourth, acute treatment before END was not included in this analysis, which might have affected the occurrence of END. A recent study suggested that dual antiplatelet therapy could improve the outcome following END (24). Fifth, we performed MRIs and MRAs using 1.5 or 3 Tesla scanner; therefore, there could be a potential bias in the comparison of the number of DWI slices between END and non-END groups.

Our study has demonstrated that the longitudinal length of an infarct lesion in patients with acute SSI without any relevant artery stenosis on MRA is an important risk factor for END development. In addition, our results suggest that performing HR MRI in subcortical infarction patients that have long longitudinal infarcted lesions without any relevant artery stenosis should be considered.

In conclusion, this study suggests that the longitudinal length of an infarct lesion is predictive of END in patients with acute SSI.

## Data Availability Statement

The raw data supporting the conclusions of this article will be made available by the authors, without undue reservation.

## Ethics Statement

The studies involving human participants were reviewed and approved by the institutional review board of Keimyung University Dongsan Medical Center, South Korea. The patients/participants provided their written informed consent to participate in this study.

## Author Contributions

SJ analyzed and interpreted the data and wrote the manuscript. S-WP, DK, HP, and S-IS analyzed and interpreted the data and revised the manuscript. J-HH designed and conceptualized the study, interpreted the data, and revised the manuscript.

## Conflict of Interest

The authors declare that the research was conducted in the absence of any commercial or financial relationships that could be construed as a potential conflict of interest.
